# Synthesis and comparative study on the structural and optical properties of ZnO doped with Ni and Ag nanopowders fabricated by sol gel technique

**DOI:** 10.1038/s41598-021-91439-1

**Published:** 2021-06-07

**Authors:** S. Al-Ariki, Nabil A. A. Yahya, Sua’ad A. Al-A’nsi, M. H. Hj Jumali, A. N. Jannah, R. Abd-Shukor

**Affiliations:** 1 Department of Physics, Thamar University, 87246 Thamar, Republic of Yemen; 2 Department of Applied Physics, Universiti Kebangsaan Malaysia, 43600 Bangi, Selangor Malaysia; 3 Faculty of Applied Sciences, Universiti Teknologi MARA, Negeri Sembilan Branch, Kuala Pilah Campus, 72000 Kuala Pilah, Negeri Sembilan Malaysia

**Keywords:** Condensed-matter physics, Nanoscience and technology, Physics

## Abstract

In this work we have tried to prepare Ni and Ag doped ZnO nanopowders using the sol gel technique. The influence of Ni and Ag (1, 3 and 5 mol.%) on the crystalline structure and optical properties of ZnO was investigated. The samples were characterized by XRD, FTIR and UV–visible spectrophotometer. XRD patterns confirmed the wurtzite formation of doped and undoped ZnO nanopowders. The average crystallite sizes of the prepared samples found from XRD were 19 nm for undoped ZnO, from 17 to 22 nm for Ni-ZnO and from 19 to 26 nm for Ag-ZnO. The average crystallite size of Ag-ZnO increased with increasing Ag contents. Different optical properties of Ni-ZnO and Ag-ZnO nanopowders were observed for different Ni and Ag content. The band gaps of Ni-ZnO and Ag-ZnO nanopowders were lower than that of the undoped ZnO (3.1 eV). The band gaps of Ag-ZnO were lower than that of Ni-ZnO. The optical properties of ZnO were enhanced by Ni (mol.%) in the UV region and by Ag (3 and 5 mol.%) in the visible region.

## Introduction

Nano-sized semiconductors have been attracting more attention due to their size-dependent electrical and optical properties. Nano-sized semiconductor may show different behaviors from the bulk semiconductor. Nano-sized metal oxide such as ZnO, CdO, SiO_2_ and PbO with different nanocomposites for example PbO–CdO^1^, CdO–SiO_2_^2^ and CdO–ZnO^3^ have been prepared by using different methods such as sol–gel and co-precipitation techniques. Nanocomposites exhibit novel optical, electrical, and magnetic properties compared with their pure metal oxide and present possibilities for several technological applications. Nano-sized oxide such as ZnO^4^, MgO, PbO^5^, NiO^6^, and Bi_2_O_3_^7^ have been added to (Bi,Pb)-2223 and exhibited enhancement in the transport critical current density (*J*_c_). The size and element of nanoparticles are important to improve in *J*_c_.

ZnO semiconductor is a promising material for applications. It is inexpensive with controllable electrical conductivity and environmentally friendly. Its properties have been extensively studied [e.g. 8]. ZnO has many applications such as for cytotoxicity^9^, antibacterial activities^10^, solar cells^11^, gas sensors^12^, and electrical and optical devices^13,14^. It has a wide band gap (3.37 eV) and large exciton binding energy (~ 60 meV), which allows efficient excitonic emission even at room temperature^15,16^.

ZnO has higher photocatalytic efficiency under UV irradiation and lower photocatalytic activity in the visible region. Therefore, significant efforts have been made to increase the ZnO activity in the visible region by metal or non-metal doped ZnO or ZnO composites^17–19^. Some elements could enhance the optical properties of ZnO while other dopants showed the opposite effect^20,21^. This implies that a significant increase in the optical properties of ZnO would still be possible.

The doping of Ni in ZnO has important influence on the optical and electrical properties which present possibilities for applications in spintronics^22,23^. The NiO doped ZnO is one of the effective methods to enhance the optical and electrical properties of ZnO. A significant decrease in resistivity of ZnO/NiO nanocomposite thin film was observed compared with undoped sample and NiO dopant is suitable for photodiodes or UV detectors. The nickel acetate tetrahydrate (C_4_H_6_NiO_4_·4H_2_O) were used as NiO source and the XRD analysis showed the NiO phase^24^. Nickel (II) chloride hexahydrate were used as Ni source and the XRD showed the Ni_2_O_3_ phase^25,26^. The nickel acetate hexahydrate (C_4_H_6_NiO_4_·6H_2_O) has also been used to prepare pure Ni_2_O_3_ nanoparticles to remove toxic Cr(VI) ion. The excess oxygen in the surrounding NiO_*x*_ produces Ni vacancies which occupy the Ni^+2^ sites and create Ni^+3^ ions^25–27^. In the reported work, the nickel nitrate hexahydrate (Ni (NO_3_)_2_·6H_2_O) was used as Ni^+2^ source.

Silver as a metal nanoparticle is a good candidate for antimicrobial and biomedical applications. Doping ZnO with Ag improved the optical properties and can be used as optoelectronic devices^9,10,18,28^. The improvement of photocatalytic activity of ZnO depends on the hindering of electron–hole recombination which is achieved by metal dopants such as Ag^29,30^. An improvement in the electrical properties of Ag doped ZnO:Al was observed due to the enhancement in the crystalline quality^31^. ZnO has also been prepared using the ultrasonic and wet chemical method^32^.

A report on Ni-doped ZnO nanoparticles with 3.9, 8.3 and 12.4 wt.% showed that Ni ions are substituted in the ZnO lattice as evidenced from X-ray diffraction and UV–visible reflectance studies^33^. In another report, X-ray diffraction and ICP-MS results confirmed the incorporation of Ni^2+^ in Cd_1−*x*_Ni_*x*_O nanoparticles with *x* = 0.047–0.163^34^.

ZnO nanopowder is a promising material for applications due to its size-dependent properties. Ni and Ag doping ZnO is one of the effective methods to enhance the optical properties of ZnO**.** It would be interesting to firstly, prepare Ni and Ag doped ZnO with particles size in the nanometer range. Then, combine ZnO with different molar ratio of Ni and Ag (1, 3 and 5 mol.%) and finally compare the properties of Ni-ZnO with Ag-ZnO nanopowders. In this work two series, i.e. Ni doped ZnO and Ag doped ZnO nanopowder samples were prepared by the sol–gel technique. The pure ZnO nanopowder was also prepared to compare with the doped samples. The influence of Ni and Ag on the crystalline structure and optical properties of ZnO was investigated. The phase and structure, and optical properties of Ni-ZnO and Ag-ZnO nanopowders were investigated by X-ray powder diffraction (XRD) and the UV–visible spectrophotometer, respectively. The samples were analyzed by FTIR spectrophotometer. The absorption, optical gap energy and crystalline size of all prepared samples were evaluated.

## Experimental details

Pure ZnO, Ni doped ZnO and Ag doped ZnO nanopowders were synthesized using the sol–gel technique as follows.

### Preparation of ZnO nanopowder

For pure ZnO nanopowder, a homogenous solution was prepared by dissolving 9.9163 g of zinc nitrate hexa-hydrate (Zn(NO_3_).6H_2_O) (FLUKA, 99%,) as zinc oxide source and 6.4041 g of citric acid anhydrous (C_6_H_8_O_7_) (HIMEDIA, 99.7%,) in de-ionized water (80 ml).

### Preparation of Ni-ZnO and Ag-ZnO nanopowders

Ni doped ZnO (Ni-ZnO) nanopowder was prepared by dissolving 9.9163 g of zinc nitrate hexa hydrate, 6.4041 g of citric acid anhydrous and nickel nitrate hexa hydrate (Ni(NO_3_)_2_.6H_2_O) (Fluka, 98%) as Ni source in de-ionized water (80 ml). Ag with ZnO (Ag-ZnO) nanopowders were synthesized using the same technique described for Ni-ZnO, except AgNO_3_ was used as Ag source instead of Ni(NO_3_)_2_.6H_2_O.

The different molar ratios (0, 1, 3 and 5 mol.%) of Ni(NO_3_)_2_.6H_2_O and AgNO_3_ were chosen to prepare Ni-ZnO and Ag-ZnO nanopowders, respectively. The prepared solutions were stirred separately by a magnetic stirrer for 3 h and heated at 42 °C. The solutions were kept at room temperature for 24 h. The resulting solutions were stirred for another 1.5 h and heated at 72 °C for 48 h. Within the third day, the reaction mixtures were stirred for 1 h and heated at 92 °C until gels were obtained. The prepared materials were dried at 120 °C for 4 h in an oven and then ground for 10 min. The resulting powders were heat-treated at 450 °C for 6 h in a furnace.

### Characterizations

The crystal structure and phase of pure ZnO, Ni-ZnO and Ag-ZnO nanopowders were investigated by X-ray powder diffraction (XD-2/XD-3 with CuK_α_ radiation; λ = 1.5406 Å.) Fourier transform infrared (FTIR) spectrophotometer of the samples was recorded using FTIR-8400S.

The lattice parameters *a*, *b* and *c* of samples were calculated by using the following equations^24,35^.

1 $$ 2d_{hkl} \sin \theta = n\lambda $$

2 $$ \frac{1}{{d_{hkl}^{2} }} = {\raise0.7ex\hbox{$4$} \!\mathord{\left/ {\vphantom {4 3}}\right.\kern-\nulldelimiterspace} \!\lower0.7ex\hbox{$3$}}(\frac{{h^{2} + hk + k^{2} }}{{a^{2} }}) + \frac{{l^{2} }}{{c^{2} }} $$

where λ is the wavelength of the XRD, *n* is the order of diffraction (for first order *n* = 1), $d_{hkl}$ is the interplanar spacing and *hkl* is the Miller indices.

The average crystallite size (*D*) of the prepared samples was determined from the XRD data using MDI Jade 5 software according to the Scherrer’s equation^24^:

3 $${D}_{hkl}=\frac{0.94\lambda }{{\beta }_{hkl}\mathrm{cos}\theta }$$

where *λ*_CuKα_ = 1.5406 Å, *β*_hkl_ is the full-width at half maximum intensity (FWHM) of the XRD peaks in radians and *θ* is the Bragg’s angle of the diffraction peak.

The optical properties of the prepared samples were investigated using UV–visible spectrophotometer (Varian, Cary 50) in the wavelength range of 200–1100 nm. The direct transitions band gap energies (*E*_*g*_) were calculated by using the Tauc's equation^36,37^

4 $$ \alpha = B\frac{{(h\nu - E_{g} )^{1/2} }}{h\nu } $$

where *α* is the optical absorption coefficient, $B$ is a constant, $h$ is the Planck constant and *ν* is the wave frequency. The *α* of the samples was determined from the following equation^38^:

5 $$ \alpha = \frac{1}{d}\ln \frac{1}{A} $$

where *A* is the absorbance and *d* is the optical path length (1 cm).

The dislocation density *δ* was measured by the following equation^10^:

6 $$ \delta = \frac{1}{{D^{2} }} $$

## Results and discussion

In this section we will discuss the XRD, FTIR and optical properties of the ZnO doped with Ni and Ag.

### XRD analysis

**Figure 1:**
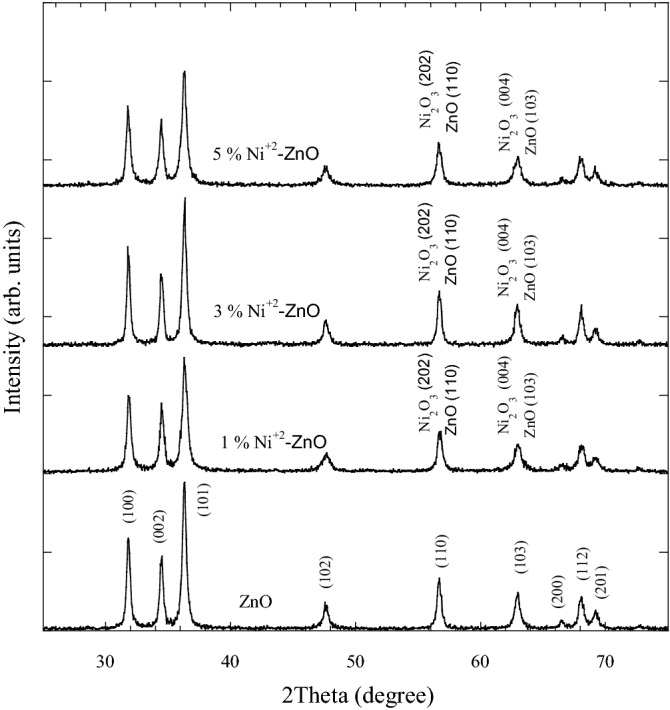
XRD patterns of pure and ZnO doped with Ni nanopowders.

Figure 1 shows the XRD patterns for ZnO and Ni-ZnO nanopowders in the range $2\theta$ = 25°–75°. The peaks of the pure sample correspond to the ZnO hexagonal wurtzite structure (JCPDS file no. 36-1451). The ZnO hexagonal phase has typical peaks with Miller indices of (100), (002), (101), (102), (110), (103), (200), (112), and (201). The diffractograms of Ni doped ZnO also maintained the hexagonal structures of ZnO and no other crystalline phase was detected. The absence of any Ni associated peaks in the diffractograms signals the successful incorporation of Ni into ZnO crystal network as also reported in a previous report^33^.

**Figure 2:**
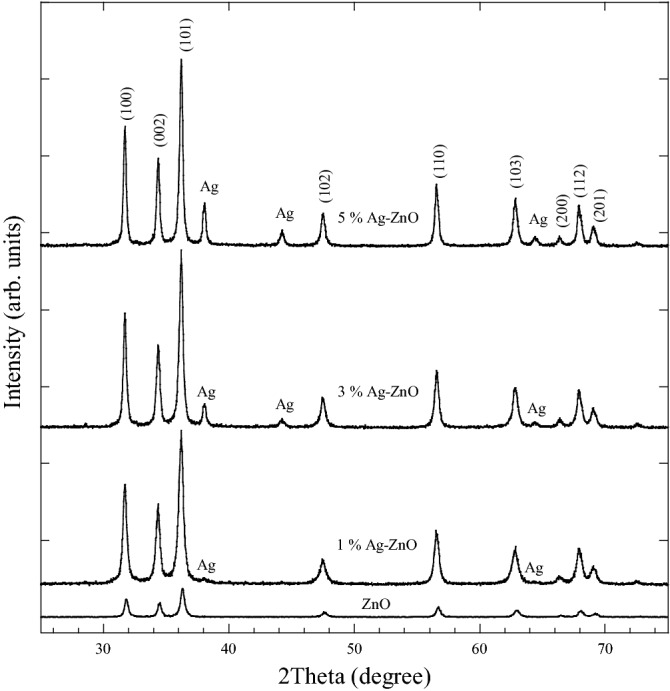
XRD patterns of pure and ZnO doped with Ag nanopowders.

XRD patterns for pure and Ag doped ZnO are shown in Fig. 2. The peaks of ZnO were verified by the presence of hexagonal structure of ZnO phase. Besides, peaks associated with cubic structures of Ag phase were observed and comparable to JCPDS file no: 04-0783. The cubic Ag phase has peaks at (111), (200), and (220). The intensity of Ag peaks increased with increasing AgNO_3_ amount. This indicated the presence of stable metallic Ag as secondary crystalline phase in the ZnO nanopowders. The presence of Ag peaks in the XRD patterns of Ag-ZnO is in agreement with results in a previous report^28^. The significantly larger radius of Ag^+1^ (0.126 nm) compared with Zn^+2^ (0.070 nm) is the possible reason for the existence of the secondary crystalline phase in the Ag-ZnO samples. The difference between radius of Ag^+1^ and Zn^+2^ limits the solubility of Ag in Zn lattice site. Tables 1 and 2 show the 2*θ*, *hkl,* phase *ID* and crystallite size (*D*) of Ni-ZnO and Ag-ZnO nanopowders as derived from XRD patterns in Figs. 1 and 2, respectively.

**Table 1:** 2*θ*, *hkl*, phase *ID* and crystallite size (*D*) of pure ZnO and Ni doped ZnO nanopowders as presented in XRD patterns analysis.

ZnO nanopowder					Ni-ZnO nanopowders							
					1 mol.%Ni		3 mol.%Ni	5 mol.%Ni		(hkl)	Phase ID	
2*θ *(deg)		(hkl)	Phase ID	D (nm)	2*θ *(deg)	D (nm)	D (nm)	D (nm)	2*θ *(deg)			
31.72		100	ZnO	21.20	31.760	19.6	22.50	19.6	31.72	100	ZnO	
34.40		002	ZnO	22.20	34.402	17.8	22.30	17.7	34.36	002	ZnO	
36.24		101	ZnO	20.00	36.279	16.8	19.50	17.5	36.24	101	ZnO	
47.64		102	ZnO	16.60	47.640	13.0	17.50	14.9	47.64	102	ZnO	
56.76		110	ZnO	19.20	56.799	17.5	20.90	17.6	56.72	202	Ni_2_O_3_ ZnO	
63.08		103	ZnO	17.00	63.081	14.4	17.70	13.6	63.12	103	ZnO	
66.68		200	ZnO	26.00	66.601	26.6	27.30	28.1	66.67	004	Ni_2_O_3_ ZnO	
68.16		112	ZnO	18.10	68.199	15.0	19.80	15.9	68.16	112	ZnO	
69.36		201	ZnO	16.40	69.320	14.5	17.70	14.0	69.32	201	ZnO	
72.88		004	ZnO	14.60	72.801	29.5	31.50	16.9	72.92	004	ZnO

**Table 2:** 2*θ*, *hkl*, phase *ID* and crystallite size (*D*) of pure ZnO and Ag doped ZnO nanopowders as presented in XRD patterns analysis.

ZnO nanopowder				Ag-ZnO nanopowders						
				1 mol.% Ag		3 mol.%Ag	5 mol.%Ag		(hkl)	Phase ID
2*θ *(deg)	(hkl)	Phase ID	D (nm)	2*θ *(deg)	D (nm)	D (nm)	D (nm)	2*θ *(deg)		
31.72	100	ZnO	21.20	31.60	21.94	26.75	29.98	31.60		
34.40	002	ZnO	22.20	34.26	21.54	24.45	29.43	34.28	002	ZnO
36.24	101	ZnO	20.00	36.12	18.57	23.03	26.84	36.12	101	ZnO
–	–	–	–	37.98	23.65	23.93	25.80	37.98	111	Ag
–	–	–	–	44.22	27.03	18.02	24.15	44.22	200	Ag
47.64	102	ZnO	16.60	47.48	18.64	19.97	21.71	47.48	102	ZnO
56.76	110	ZnO	19.20	56.60	19.10	22.51	27.49	56.62	110	ZnO
63.08	103	ZnO	17.00	62.94	15.49	19.69	23.72	62.94	103	ZnO
–	–	–	–	64.52	21.53	22.83	21.95	64.52	220	Ag
66.68	200	ZnO	26.00	66.46	15.43	23.86	27.88	66.56	200	ZnO
68.16	112	ZnO	18.10	68.06	22.30	21.13	24.68	68.06	112	ZnO
69.36	201	ZnO	16.40	69.18	16.80	20.91	22.38	69.2	201	ZnO
72.88	004	ZnO	14.60	72.70	17.27	21.65	27.21	72.74	004	ZnO

The average lattice parameters for pure ZnO were *a* = *b* = 3.2547 Å, *c* = 5.2103 Å, and *c/a* = 1.6009 (Table 3). These values are consistent with the JCPDS file no. 36–1451 (*a* = *b* = 3.2535 Å, *c* = 5.2151 Å, and *c/a* = 1.60292). The was a slight variation of the lattice parameters with the presence of Ni and Ag from that of pure ZnO. That may be due to the successful dopant incorporation in Ni-ZnO and existence of microstructural strain from defects in Ag-ZnO^35,39^.

**Table 3:** The average crystalline size, lattice parameters, average FWHW and dislocation density of pure ZnO, Ni doped ZnO and Ag doped ZnO nanopowders.

Sample	D (nm) Avg	FWHM Avg (deg)	Optical Band gap (eV)	$\delta$ (nm^−2^)	*a* = *b *(Å)	*c *(Å)	*c*/*a*
ZnO	19.13	0.4259	3.10	0.0027	3.2547	5.2103	1.6009
1 mol.% Ni-ZnO	18.47	0.4581	3.05	0.0029	3.2507	5.2096	1.6026
3 mol.% Ni-ZnO	21.67	0.3793	2.95	0.0021	3.2547	5.2099	1.6007
5 mol.% Ni-ZnO	17.77	0.4697	3.00	0.0032	3.2549	5.2156	1.6024
1 mol.% Ag-ZnO	19.95	0.4214	2.90	0.0025	3.2667	5.2305	1.6012
3 mol.% Ag-ZnO	22.21	0.3612	2.00	0.0020	3.2668	5.2276	1.6002
5 mol.% Ag-ZnO	25.63	0.3134	1.80	0.0015	3.2668	5.2277	1.6003

**Figure 3:**
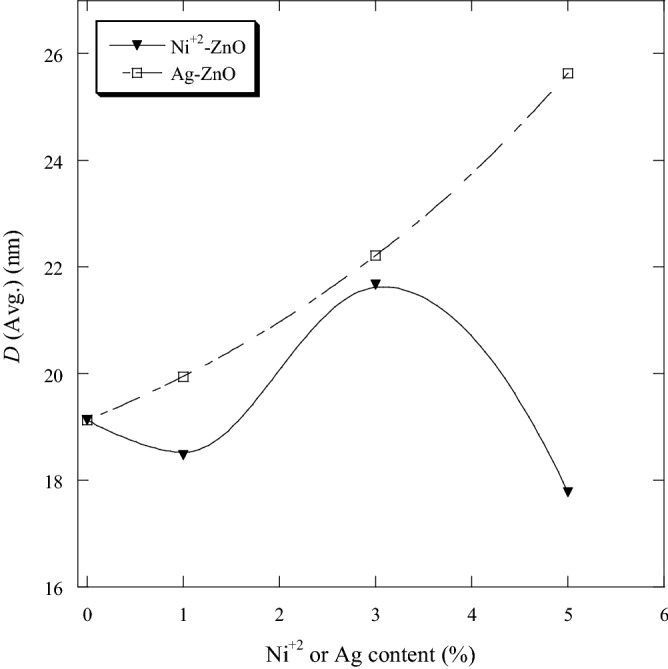
The average crystallite sizes of pure and ZnO doped as a function of Ni and Ag contents.

From the XRD data, the crystallite sizes (*D*) of Ni-ZnO and Ag-ZnO nanopowders were estimated from FWHM of all the peaks using the Scherrer’s equation (Eq. 3). For comparative purpose, Fig. 3 shows the average crystallite size of prepared samples as a function of Ni and Ag contents. The average crystallite sizes of the prepared samples were found to be in the nanometer range of 17–22 nm for Ni-ZnO and 19–26 nm for Ag-ZnO. By comparison, the average crystallite size of pure ZnO was 19 nm. Doping ZnO with 1 and 5 mol.% Ni produced broader XRD peaks and hence lower average crystallite sizes. However, ZnO doped with 3 mol. % Ni showed narrower peaks than pure ZnO suggesting the increment in average crystallite size and reduction in dislocation density^35^. The crystallite sizes of Ag-ZnO increased with increasing Ag content, whereas the average FWHM was decreased with increase in Ag. The crystallite sizes of Ag-ZnO were in good agreement with the results in a previous report^28^. The average crystallite size, lattice parameters, average FWHM and dislocation density of pure ZnO and doping ZnO with Ni and Ag nanopowders are shown in Table 3.

### FTIR spectroscopy

**Figure 4:**
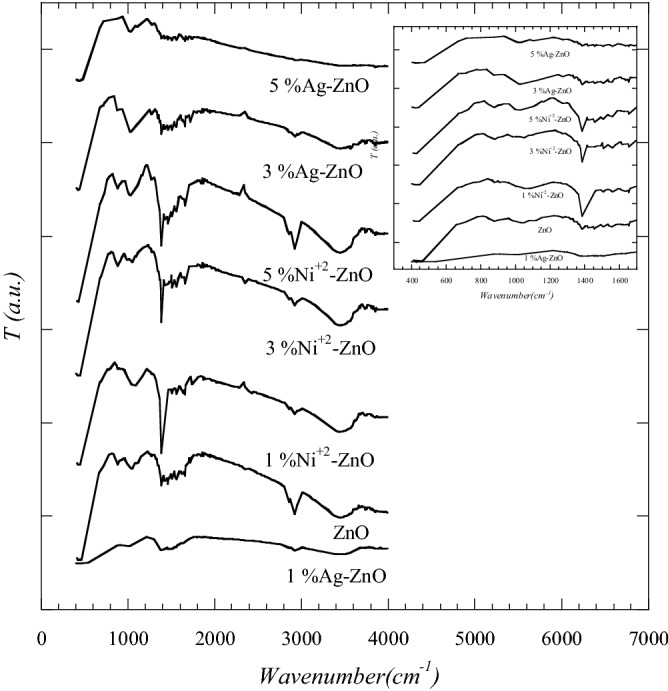
FTIR spectrophotometer of pure, Ni doped ZnO and Ag doped ZnO nanopowders.

Pure ZnO and Ni and Ag with ZnO nanopowders were investigated by Fourier transform infrared (FTIR) spectroscopy in the range from 400 up to 4000 cm^−1^. The FTIR spectra of pure and doped ZnO samples exhibited various absorption bands (Fig. 4). In all samples, the absorption bands in the range between 400 and 467 cm^−1^ corresponds to the vibrations of Zn–O. Absorption band observed in the region between 3396 and 3502 cm^−1^ corresponds to the vibrations of O–H which showed the existence of humidity in the samples due to water absorption. The band near 2900 cm^-1^ corresponds to the stretching and vibrations of C–H absorption while band observed in the region between 1100 and 1600 cm^−1^ corresponds to the OH^36^. A slight shift to lower frequencies was observed in the bands of doped samples compared to that of undoped sample as shown in the inset of Fig. 4. This slight shift could be due to the doping of ZnO with Ni and Ag which changed the bond length. The FTIR spectra for Ni-ZnO samples exhibited weak band near 700 cm^−1^ which may be due to Ni–O bending vibrations. For ZnO with Ag samples, the weak band near 900 cm^−1^ may correspond to the stretching and vibrations of Ag and ZnO^10^.

### Optical properties

**Figure 5:**
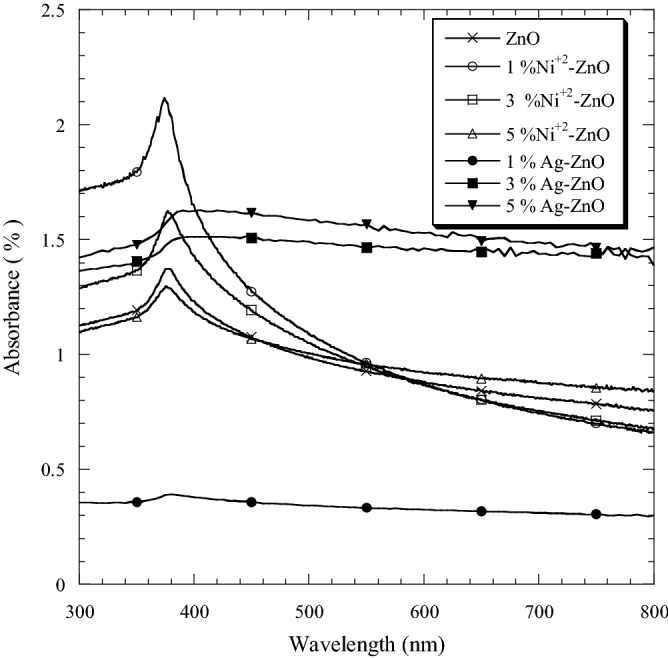
The optical absorption spectra of pure, Ni doped ZnO and Ag doped ZnO nanopowders.

The optical absorptions of the samples were determined at room temperature using the UV–visible spectrophotometer within the wavelength range of 300–800 nm. Figure 5 shows the absorption spectra for Ni–ZnO and Ag–ZnO nanopowders with 0, 1, 3 and 5 mol.% of Ni and Ag. The absorptions spectra of Ni–ZnO and Ag–ZnO have almost similar behavior to that of pure ZnO. The doping of Ni and Ag may influence the optical absorption of ZnO. Pure ZnO nanopowders exhibited absorption edge in the range of 376 nm, which relates to the UV region. The absorption edges of doped ZnO with molar ratios of 1, 3 and 5 mol.% Ni were observed at 374 nm, 377 nm and 376 nm, respectively. The absorption edges for Ag-ZnO samples with molar ratios of 1, 3 and 5 mol.% Ag were observed at 379 nm, 387 nm and 390 nm, respectively. It appears that the absorbance in the UV band of both samples was higher, except for 1 mol.% of Ag in comparison with the pure ZnO. Among Ni–ZnO, the highest absorption was in small amount of Ni (1 mol.%) within the wavelength range of 300–550 nm. In Ag–ZnO series, a small amount of Ag (1 mol.%) exhibited a weak absorbance. However, the higher amount (5 mol.%) presented the highest absorption between 300 and 800 nm. This indicates that the absorption bands of Ag-ZnO (3 and 5 mol.%) in the visible region were wider compared to the pure and Ni doped ZnO. The sample with molar ratio of 1% Ni exhibited a strong absorption in the UV band and weak absorbance in the visible region. Therefore, it can be potentially used as sunscreens in commercial products^21^. In wavelength larger than 400 nm, the optical absorption was the highest with 3 and 5 mol.% Ag compared to other samples. For future work, these samples can be used as an efficient photocatalyst under irradiation with visible light of wavelength larger than 400 nm.

**Figure 6:**
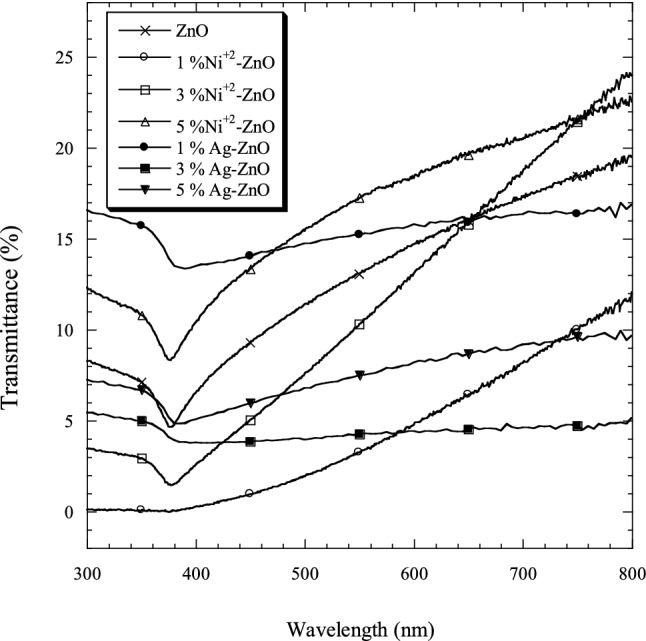
The optical transmittance spectra of pure and ZnO doped with Ni and Ag nanopowders.

Figure 6 shows the transmittance spectra of Ni–ZnO and Ag–ZnO nanopowders with molar ratios of 0, 1, 3 and 5 mol.% Ni and Ag within the wavelength range of 300–800 nm. It can be seen that a small amount of Ni (1 mol.%) decreased the optical transmission, whereas a higher amount (5 mol.% Ni) was highly transparent in the UV and visible regions as compared with the pure ZnO.

In Ag–ZnO samples, a small amount of Ag (1 mol.%) doped ZnO was highly transparent between 300 and 740 nm in comparison to the pure ZnO. Doping ZnO with 3 and 5 mol.% Ag decreased the optical transmission as compared to the pure ZnO. Ni–ZnO showed higher transparency in the visible regions greater than 480 nm and the lowest transparency in wavelength smaller than 480 nm in comparison to the pure and Ag doped ZnO. By comparing all samples, Ag–ZnO exhibited the highest transparency within wavelength smaller than 440 nm.

**Figure 7:**
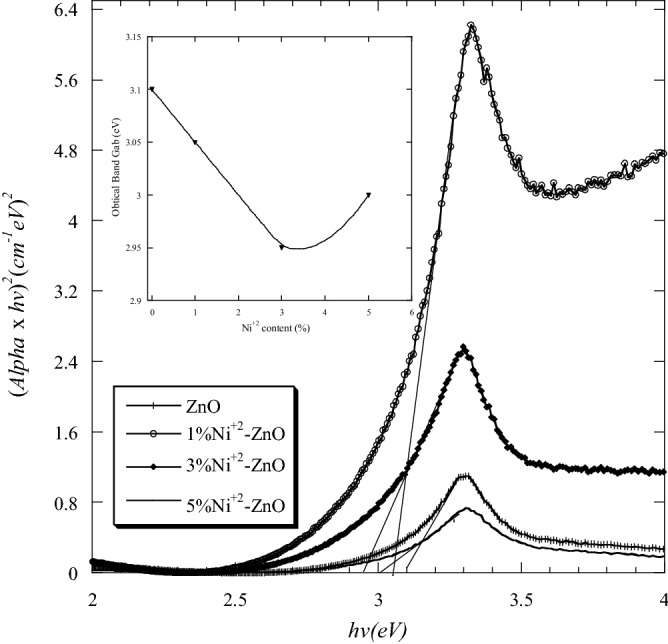
$(\alpha h\nu )^{2}$ versus the photon energy ($h\nu$) of pure and Ni doped ZnO nanopowders. The inset is the energy gap of samples as a function of Ni content.

From Eq. (4) and plotting $(\alpha h\nu )^{2}$ versus the photon energy ($h\nu$), the value of energy gap (*E*_*g*_) can be found by extrapolating the intercept of the curve to zero absorption in the photon energy axis. The band gap energy of pure ZnO nanopowder (3.1 eV) was less than that of bulk ZnO (3.37 eV). This reduction in the energy band gap agrees with those reported in the literature^10,37^. The band gap energies of Ni–ZnO and Ag-ZnO nanopowders were smaller than that of pure ZnO nanopowder which may be due to the effect of Ni and Ag concentrations. The band gap energies of Ni–ZnO nanopowders varied between 3.1 and 2.95 eV (Fig. 7). They were 3.05 eV, 2.95 eV and 3 eV for 1, 3 and 5 mol.% Ni, respectively. The inset of Fig. 7 shows the decreasing of energy gap of Ni-ZnO nanopowders compared to the pure ZnO.

**Figure 8:**
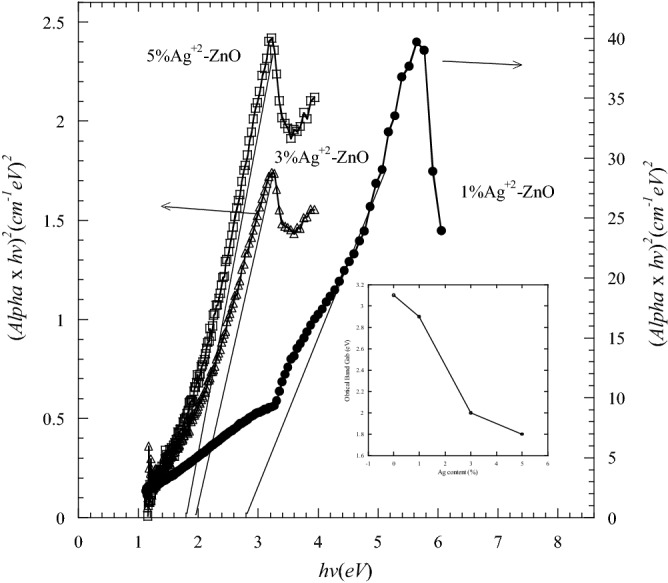
$(\alpha h\nu )^{2}$ versus the photon energy ($h\nu$) of pure and Ag doped ZnO nanopowders. The inset is the energy gap of samples as a function of Ag content.

The band gap energies of Ag-ZnO nanopowders were reduced as compared with the pure ZnO (Fig. 8). The band gap decreased with increasing Ag content. They were 2.8 eV, 2 eV and 1.8 eV for 1, 3 and 5 mol.% Ag, respectively. The band gap energies of Ag-ZnO are shown in the inset of Fig. 8.

**Figure 9:**
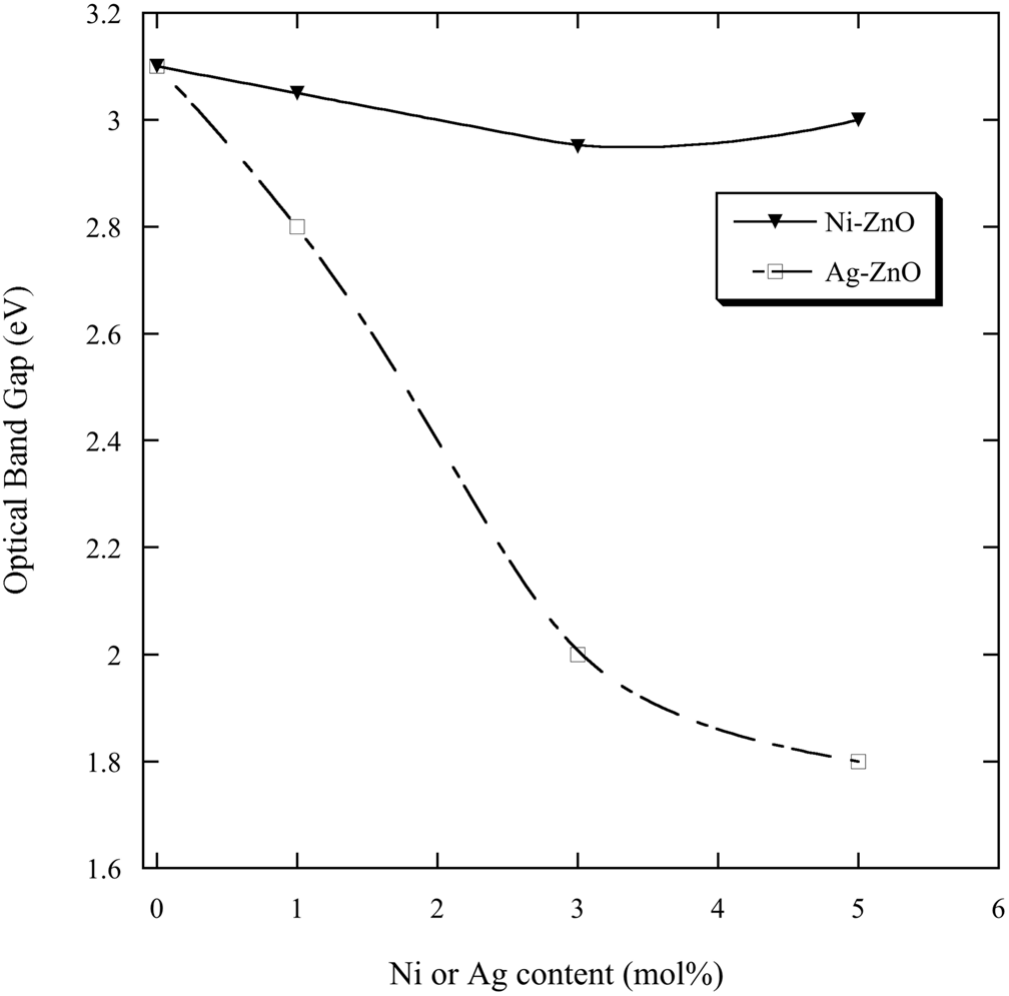
The energy gaps of samples as a function of Ni and Ag contents.

For comparison purpose, Fig. 9 and Table 3 show the values of band gap energies of the samples as a function of Ni and Ag contents. The band gap energies of Ag–ZnO were smaller compared with that of pure ZnO and Ni-ZnO samples. The decrease in the band gaps of the samples is useful in optoelectronic devices^40^. There are several factors for the decrease in the optical energy band gap such as grain size, carrier concentration, structural parameters and lattice strain and existence of defects or impurities^38,39^.

## Conclusions

The effect of Ni and Ag on the structure and optical properties of ZnO has been investigated. Pure ZnO and ZnO containing different molar ratio of Ni and Ag (0, 1, 3 and 5 mol.%) were prepared by the sol gel technique. The crystallite sizes of all samples were estimated by using the Scherrer’s equation and were in the nanometer range. The optical properties of the samples were determined by UV–visible spectrophotometer. Ni and Ag had different effects on the structure and optical properties of ZnO. The average crystallite size was 19 nm for pure ZnO. A slight decrease in the average crystallite size of Ni-ZnO was observed in comparison to the pure ZnO. Ni-ZnO also showed a smaller crystallite sized compared to Ag-ZnO. The average crystallite sizes were between 17 and 22 nm for Ni–ZnO and between 19 and 26 nm for Ag-ZnO. The band gap energy of pure ZnO was 3.1 eV. The doped samples exhibited better optical properties in comparison with pure ZnO. A slight decrease in the band gap energies of Ni-ZnO was observed in comparison to the pure ZnO. Ag-ZnO exhibited a higher decrease in the band gap energies than Ni-ZnO. Ni-ZnO showed better absorption in the UV band whereas Ag-ZnO exhibited better absorption in the visible light. The improvement of optical properties of ZnO depends on the type and amount of metal oxide dopants.
